# Effects of Irrigation and Nitrogen Fertilizer Input Levels on Soil NO_3_^−^-N Content and Vertical Distribution in Greenhouse Tomato (*Lycopersicum esculentum* Mill.)

**DOI:** 10.1155/2016/5710915

**Published:** 2016-08-23

**Authors:** Xiukang Wang, Yingying Xing

**Affiliations:** ^1^College of Life Science, Yan'an University, Yan'an, Shaanxi 716000, China; ^2^Key Laboratory of Agricultural Soil and Water Engineering in Arid and Semiarid Areas of Ministry of Education, Northwest A&F University, Yangling, Shaanxi 712100, China

## Abstract

The purpose of this study is to investigate the interactions between irrigation and fertilizer treatments on soil NO_3_^−^-N content and vertical distribution under drip fertigation in greenhouse tomatoes. Randomized block design with three replications and the treatments consisting of three levels of irrigation and three levels of N fertilizer were used. Three irrigation levels were W1 (100%  ET_0_), W2 (75%  ET_0_), and W3 (50%  ET_0_) and fertilizer levels were F1 (N240–P_2_O_5_120–K_2_O150 kg hm^−2^), F2 (N180–P_2_O_5_90–K_2_O112.5 kg hm^−2^), and F3 (N120–P_2_O_5_60–K_2_O75 kg hm^−2^). The result demonstrates that dynamics of soil NO_3_^−^-N and its response to drip fertigation and levels of N moved toward the fore soil moist, and the average soil NO_3_^−^-N content with W3 treatment was 1.23 times higher than that of the W1 treatment in 0–60 cm at 43 days after transplanting. The negative correlation between N use efficiency and levels of fertilizer N and the N recovery efficiency was increased with increases of N fertilizer application. The fertilizer nitrogen rate greatly significantly influenced soil NO_3_^−^-N content. Avoiding N leaching through controlled matching N fertilizer application and controlled irrigation to tomato N demand is the key to maintain crop yield and improve N use efficiency.

## 1. Introduction

Fertilizer application is one of the most effective and practical ways to control and improve yield and nutritional quality of crops for human consumption. In the current food production scenario across major cropping systems of the world, crop yield is limited more by availability of nitrogen (N) and water resources rather than by the crop genetics [1]. Although the soil nutrient input on farmland is the need to make their crops grow better, soil nutrient loss from agricultural areas is a major source of pollution for freshwater and coastal ecosystems [2]. The supply of N to a crop is determined by the amount of plant-available N in soil at the growing time, N released during the growing season through mineralization of soil organic matter, and N applied as organic or inorganic fertilizer [3]. In some experiments, late N application showed no effect on grain yield, but increased grain protein, as did increasing the rate of late applied N. Many other studies have shown the relationships between increasing soil NO_3_^−^-N concentration and increasing crop grain yield and protein concentration [4–7]. Hence, very often, they are applied at higher doses than required. However, there has been growing concern over the potential environmental impact of higher doses of inorganic N fertilizer use in crop fields.

Residual soil NO_3_^−^-N is seldom considered as a source of N. Indiscriminate use of N fertilizer without considering the contributions of residual soil NO_3_^−^-N leads to low crop uptake and thus further growth in the N residual populations, which leaches down to the lower depths and ground water. Then, the environmental consequences of N use in food production are strongly related to crop N recovery. The N fertilizer that was used in the global mountainous region of crop production is about 75%; however, the N absorption content of crop is commonly about 50% at this field scale [8]. The surplus of N may accumulate in soils or be lost in air, groundwater, and surface water via various pathways. The surplus of N was a rapid decrease in the form of gaseous dinitrogen, nitrous oxide, and nitric oxide, volatilization of ammonia, leaching of nitrate, and runoff and erosion [9].

N is an essential element for plant growth and a key element of agricultural input [10–12]. Rapid increases of crop yields became possible when synthetic N fertilizer became available after the discovery of the Haber-Bosch process in the early 20th century [13, 14]. However, increased use of N fertilizers has also led to increased N losses from agroecosystems, especially since the 1950s [15]. The N fertilizer distribution across the globe is very uneven. In some areas, the N fertilizer is used excessively and leads to N pollution, causing a host of problems for human and ecological health. In the other parts of the world, suffer from the degeneration of soil fertility, the reduction of crop yield, or other consequences exist.

The coupling effect of water and fertilizer is not distinct and more fertilizers can be used to compensate for the shortage of water under limited water resources. Meanwhile, through the coupling of water and fertilizer, N affects water consumption. But we still have limited information about the effects of different measures of controlling the supply of water and fertilizer on nutrient uptakes, accumulations, and translocation. Thus, the present study aim was to focus on the relationships between the irrigation and N fertilizer input levels and the soil NO_3_^−^-N content and vertical distribution.

## 2. Materials and Methods

### 2.1. Experimental Site

The experiment was conducted at the Key Laboratory of Agricultural Soil and Water Conservation Engineering in Arid Areas, the Ministry of Education (34°20′N, 108°04′E and altitude 521 m), Shaanxi Province, China. The climate is a warm temperate, semiarid climate zone with a mean annual air temperature of 13°C. The mean annual precipitation is 645 mm, of which approximately 70% falls between July and September, and the average annual evaporation is 1500 mm. The length, span, and height of the experimental greenhouse are 76 m, 7.5 m, and 2.8 m, respectively. The soil is heavy loam in texture according to the USDA texture classification system, which is derived from loess with a deep and even soil profile.

The topsoil (0–80 cm) in the greenhouse has a field capacity of 23–25% and a wilting moisture content of 8.5% (all quality water contents). Soil sample was dried at room temperature (75°C) in the laboratory to a constant weight and sieved (2 mm) to eliminate coarse soil particles. Soil acidity (pH) was measured in an aqueous soil extract in deionized water (1 : 2.5 soils : water). Bulk density was measured by the core method, using cores that measured 3 cm in diameter, 10 cm in length, and 70.68 cm^3^ in volume. Field capacity at 33 kPa was determined using a pressure-membrane extraction apparatus. Soil organic matter was determined using the Walkley-Black method. Soil sample was dried at room temperature (75°C) in the laboratory to a constant weight and sieved (2 mm) to eliminate coarse soil particles. Soil bulk density was measured by the core method, using cores that measured 3 cm in diameter, 10 cm in length, and 70.68 cm^3^ in volume. Field capacity at 33 kPa was determined using a pressure-membrane extraction apparatus. Soil organic matter was determined using the Walkley-Black method. The contents of organic matter, available N, available P, and available K (*N* = 3) were measured using a spectrophotometer (UV-VIS 8500 II, China), which are shown in Table 1.

### 2.2. Experimental Design

In this experiment, nine treatments were designed with three different irrigation levels (W1: 100%  ET_0_; W2: 75%  ET_0_; and W3: 50%  ET_0_) and fertilizer levels (F1: N240–P_2_O_5_120–K_2_O150 kg hm^−2^; F2: N180–P_2_O_5_90–K_2_O112.5 kg hm^−2^; and F3: N120–P_2_O_5_60–K_2_O75 kg hm^−2^). For the control (CK), without fertilizer application and the irrigation levels of W2, the experiment was organized using a randomized block design with three replications, and each plot was 6 m long, 3.75 m wide, and 22.5 m^2^ in the greenhouse. There were 30 divided and ridged experimental plots, which were divided by a water barrier sheet.

The tomatoes (*Lycopersicum esculentum *Mill., cv. “Jinpeng 10”) were planted on April 5, 2012, and March 31, 2013. The furrow-film mulch was cultivated by the local, traditional, planting patterns and ridging in one drip tube with two-line tomato seedlings layout, spaced 50 cm apart, with a 45 cm planting distance and 78 plants in each experimental plot. Drip fertigation was performed with a fertilizer of urea (46% N), diammonium phosphate (44% P_2_O_5_), and potassium chloride (60% K_2_O). The fertigation equipment used hydraulic proportional pump to control the fertilizer amount, which was composed of a water source, water pump, rotor meter, and a scale fertilizer pump and conveyance pipeline system. The drip line consisted of an insert cylinder head drip irrigation pipe, with an inner diameter of 8 mm, a drop head span of 30 cm, a head flow of 2 L h^−1^, and drip irrigation operating pressure of 0.3 MPa.

Irrigation treatments were initiated using the surface drip irrigation system during planting, and the irrigation amount was 40 mm. According to the daily ET_0_ and irrigation management level, the irrigation amounts of W1, W2, and W3 were 262.00, 206.60, and 151 mm in 2012, 279.54, 219.66, and 159.77 mm in 2013. The fertilizers of N, P_2_O_5_, and K_2_O were applied five times at 10 days after planting, 25 days after planting, the first fruit enlargement period, the second fruit enlargement period, and the third fruit enlargement period, and the fertilization ratio was 1 : 1 : 2 : 2 : 2. The water meter and hydraulic proportion fertilization pump accurately controlled the irrigation water and fertilizer levels.

### 2.3. Sampling and Measurement

During the growing season, the soil NO_3_^−^-N content (*N* = 3) was measured using a spectrophotometer (UV-VIS 8500 II, Shanghai Scientific Instrument Co., Ltd., China), and the depth interval spacing was 10 cm (from 0 to 60 cm). The soil NO_3_^−^-N content was measured below the emitter, and the distance from sample point to emitter was 10, 20, and 30 cm. The measurements were performed at 25, 43, 59, and 117 days after transplanting.

As the soil sample collections finish, the measurement was carried out. First, 0.5 g of fresh soil was placed in a 100 mL triangular flask. Then, 50 mL of a 2-mol L^−1^ potassium chloride solution was added. The solution was shaken for half an hour until uniformity was reached. Then, the solution was filtered, and 5 mL was placed in a spectrophotometer and examined at a wavelength of 210 nm [16]. The soil NO_3_^−^-N content was determined using colorimetric analysis. Total N was determined after incineration at 900 C in a VarioMAX CN analyzer and determined by a Thermal Conductivity Detector (TCD) [17].

At final harvest, two rows of tomatoes (70 cm wide, 600 cm long) in the middle of each treatment for the three replicates were hand-harvested, and the tomato fresh yield was determined based on electronic weighing.

### 2.4. Statistical Analysis and Calculation

The effective residual of soil NO_3_^−^-N content (from 0 to 60 cm), N absorption contents by plant, % fertilizer N recovery efficiency, and % fertilizer N use efficiency were calculated, using the following equations [18, 19]:

(1) Effective residual of soil NO_3_^−^-N content (N_r_, kg hm^−2^) = *C*_1_ (mg kg^−1^) × *h* (cm) × *ρ* (g cm^−3^) × 10 × 0.01, where *C*_1_ is soil NO_3_^−^-N content, *h* is soils thickness, and *ρ* is soil bulk density.
(2) The N absorption contents (N_s_, kg hm^−2^) = *C*_2_ (mg kg^−1^) × FW (kg) × 10000 (m^2^ hm^−2^)/area harvest (m^2^) × SDW (kg)/SFW (kg), where *C*_2_ is plant total N content, FW is sample fresh weight per area harvested, SDW is subsample dry weight, and SFW is subsample fresh weight.
(3) % Fertilizer N recovery efficiency = ((N_r_ − N_i_)+(N_s_ − N_c_))/N_a_ × 100, where N_r_ is the effective residual of soil NO_3_^−^-N content (kg hm^−2^), N_i_ is initial effective accumulation of soil NO_3_^−^-N from 0 to 60 cm (kg hm^−2^), N_s_ is the N absorption contents (kg hm^−2^), N_c_ is N absorption contents of control treatment per area harvested (kg hm^−2^), and N_a_ is N fertilizer application (kg hm^−2^).
(4) % Fertilizer N use efficiency = N_s-f_/N_a_ × 100, where N_s-f_ is the N absorption contents in fruit yield of tomato (kg hm^−2^) and N_a_ is N fertilizer application (kg hm^−2^).

Analysis of variance was conducted on soil NO_3_^−^-N content using a two-way analysis of variance (SAS GLM procedure version 9.2, SAS Institute Ltd., North Carolina, USA). Duncan's multiple range tests were considered significant when *P* < 0.05.

## 3. Results

### 3.1. Soil NO_3_^−^-N Content

**Figure 1:**
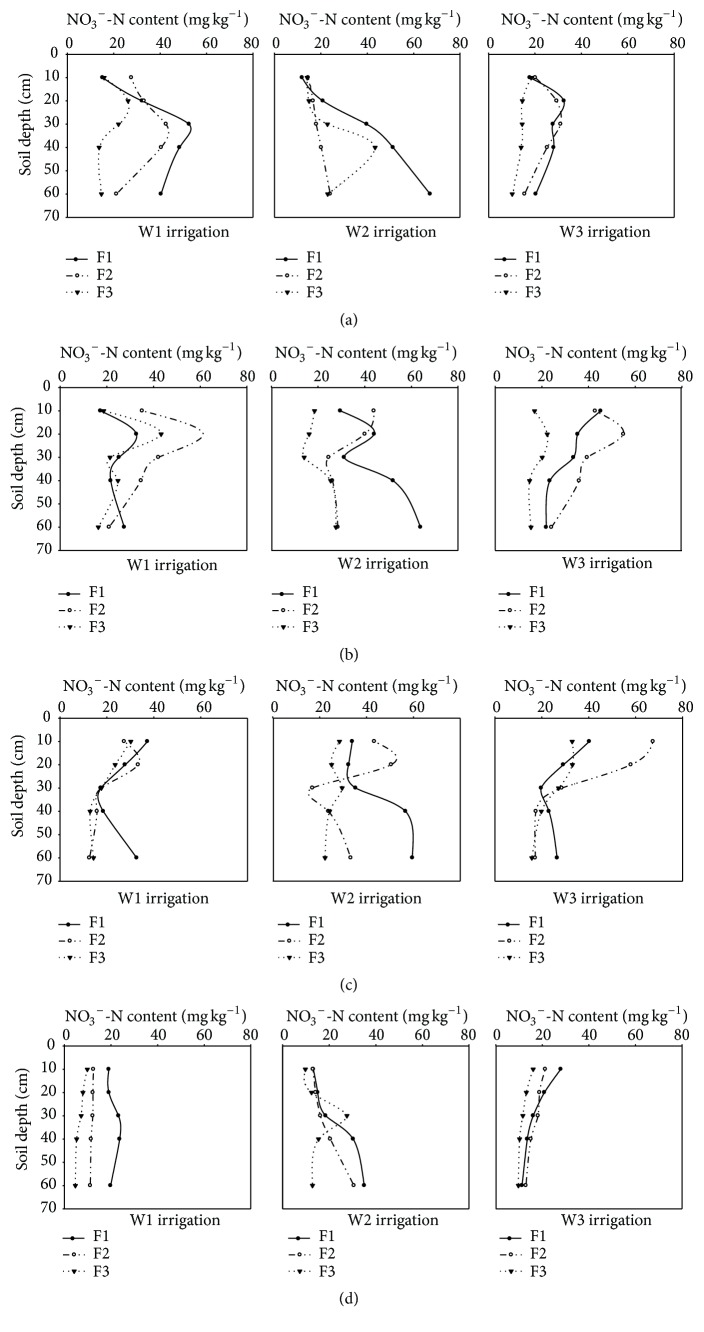
The effects of different irrigation and fertilization on soil NO_3_^−^-N content were varied by soil depth in 2012: (a) 25 days after transplanting, (b) 43 days after transplanting, (c) 59 days after transplanting, and (d) 117 days after transplanting. W1: 100%  ET_0_: W2: 75%  ET_0_, and W3: 50%  ET_0_; F1: N240–P_2_O_5_120–K_2_O150 kg hm^−2^; F2: N180–P_2_O_5_90–K_2_O112.5 kg hm^−2^; and F3: N120–P_2_O_5_60–K_2_O75 kg hm^−2^.

The variation of soil NO_3_^−^-N contents in root zone on different irrigation and N fertilizer application are shown in Figure 1. The soil NO_3_^−^-N contents ranged from 4.72 to 67.47 mg kg^−1^ in all the treatments. In the whole growth period, the soil NO_3_^−^-N concentration of root zone showed an increasing trend first and then a downward trend, but the variation rate is inconsistent.

At 25 days after transplanting, the soil NO_3_^−^-N contents in F1 treatment were significantly higher than F3 (Figure 1(a)). The soil NO_3_^−^-N contents ranged from 13.70 to 52.45 mg kg^−1^ in W1 treatment, and the soil NO_3_^−^-N content was increased with soil depth in 0–30 cm except W3 treatment.

In 43 days after transplanting, the vertical distribution of soil NO_3_^−^-N contents was similar to 25 days after transplanting, and the average soil NO_3_^−^-N content in F2 was higher than F1 and F3 (Figure 1(b)). The high concentration of soil NO_3_^−^-N content was mainly distributed at surface layer, which was nearly at the soil depth of about 10 to 20 cm. The horizontal distribution of soil NO_3_^−^-N content (20−40 cm) was obviously higher than that in the root absorption area (0−20 cm). The average soil NO_3_^−^-N content with W3 treatment was 1.23 times higher than the W1 treatment in 0−60 cm.

In 59 days after transplanting, the soil NO_3_^−^-N content in F1 treatment was increased from 30 to 60 cm at irrigation levels of W1, but the irrigation levels of W3 were decreased (Figure 1(c)). Overall, the soil NO_3_^−^-N content showed a decreased tendency in W3 treatment from 30 to 40 cm, but the tendency was increasing obviously in W2 treatment.

**Figure 2:**
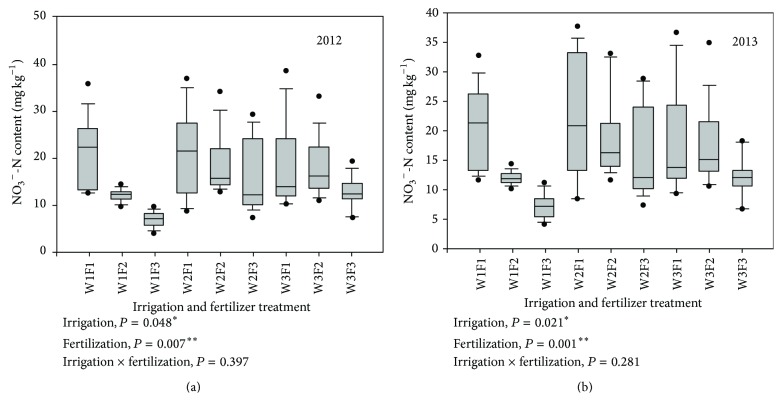
The average soil NO_3_^−^-N contents of 0–60 cm soil profile after harvesting. W1: 100%  ET_0_, W2: 75%  ET_0_, and W3: 50%  ET_0_; F1: N240–P_2_O_5_120–K_2_O150 kg hm^−2^; F2: N180–P_2_O_5_90–K_2_O112.5 kg hm^−2^; and F3: N120–P_2_O_5_60–K_2_O75 kg hm^−2^.

Compared to the former period, the values of soil NO_3_^−^-N content were decreased obviously in 117 days after transplanting (Figure 1(d)). The soil NO_3_^−^-N content ranged from 4.72 to 27.94 mg kg^−1^, and the tendency was decreased with soil depth increasing. There was a positive correlation between the fertilizer application rate and soil NO_3_^−^-N content. At final harvest, a statistically significant difference in soil NO_3_^−^-N content was found between different levels of irrigation and fertilizer inputs, but the interaction of irrigation and fertilizer had no significant effect on soil NO_3_^−^-N content in the two consecutive years (Figure 2).

### 3.2. The Vertical Distribution of Soil NO_3_^−^-N

**Figure 3:**
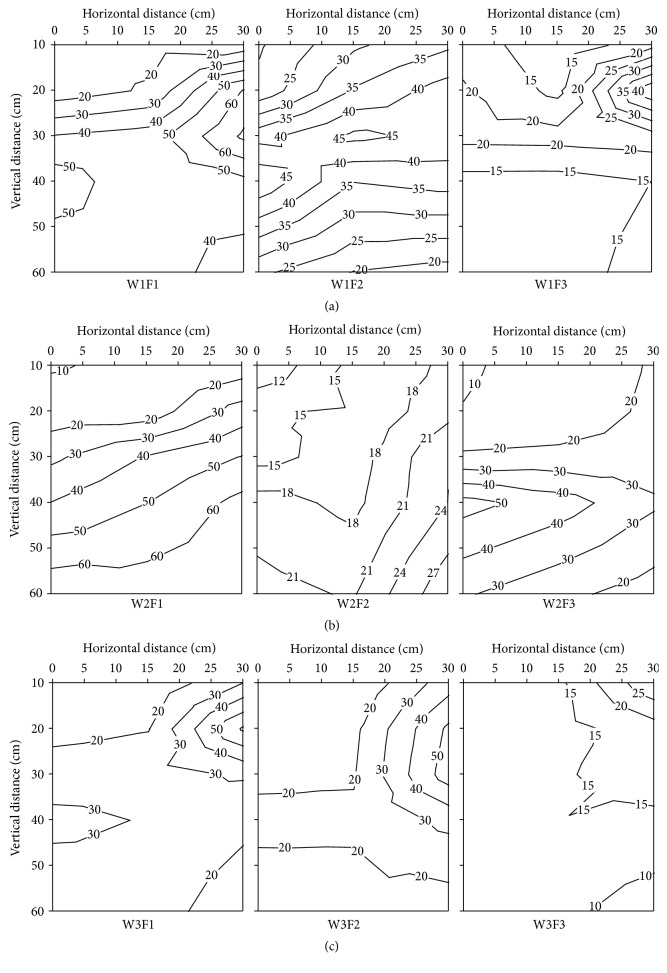
The effects of different irrigation and fertilization on vertical distribution of soil NO_3_^−^-N content in root zone soil of different treatment at 25 days after transplanting in 2012. W1: 100%  ET_0_, W2: 75%  ET_0_, and W3: 50%  ET_0_; F1: N240–P_2_O_5_120–K_2_O150 kg hm^−2^; F2: N180–P_2_O_5_90–K_2_O112.5 kg hm^−2^; and F3: N120–P_2_O_5_60–K_2_O75 kg hm^−2^.

The variations of soil NO_3_^−^-N contents in root zone soil of different treatment on 25 days after transplanting are shown in Figure 3. The vertical distribution of soil NO_3_^−^-N contents ranged from 7.29 to 71.98 mg kg^−1^, and the obvious accumulation area of soil NO_3_^−^-N contents was present in the upper 30 cm of root system in F1 treatment and 20 cm of root system in F3 treatment. The soil NO_3_^−^-N contents increase with the soil depth increases nearby the emitter and ranged from 0 to 15 cm in F1 treatment, but the soil NO_3_^−^-N increased at first and then decreased as the soil depth increases in F2 and F3 treatment (Figure 3(a)). The results show that the effects of fertilizer level were significant on vertical distribution of soil NO_3_^−^-N contents in W2 treatment, and the degree of impact of soil NO_3_^−^-N contents distribution followed the order F1 > F3 > F2. The soil NO_3_^−^-N contents increases with the increases of soil depth and the higher increase rate of soil NO_3_^−^-N contents in F1 treatment, compared to the F2 treatment. In F3 treatment, the soil NO_3_^−^-N contents increase in the topsoil layers (0−30 cm) and decrease from 40 cm to 60 cm; at the same time, there was an obvious accumulation area of soil NO_3_^−^-N contents in 40 cm (Figure 3(b)). The N fertilizer application rate is one of the most important factors affecting the vertical distribution of soil NO_3_^−^-N contents; the average soil NO_3_^−^-N contents in F1 and F2 treatment was significantly higher, that is, 43.33% and 40.99%, than the F3 treatment. The accumulation area of soil NO_3_^−^-N contents appeared at the below of root system (Figure 3(c)).

**Figure 4:**
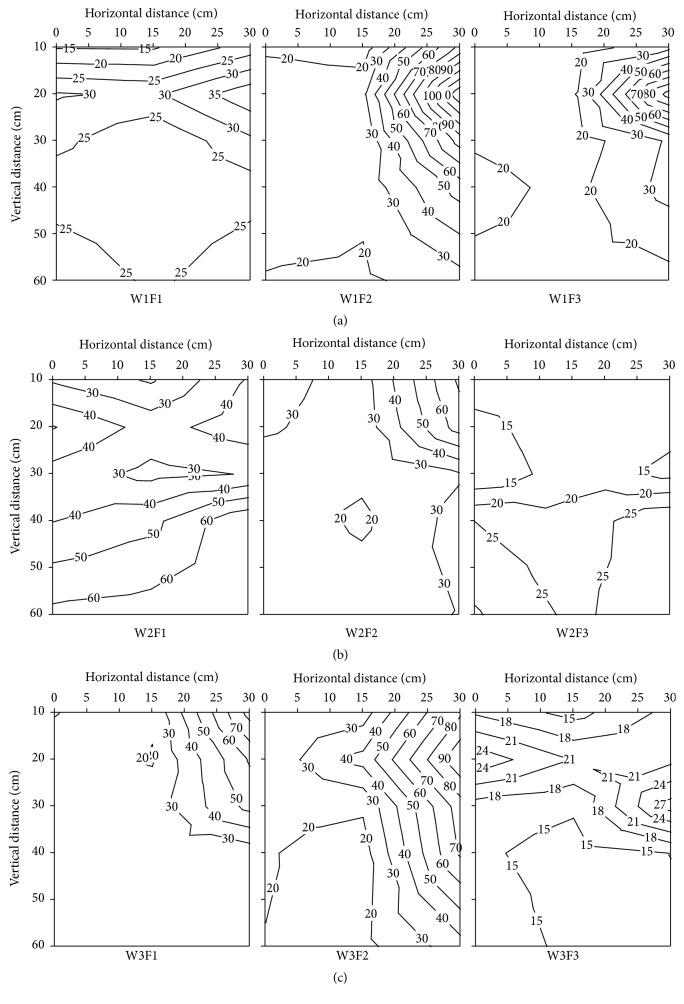
The effects of different irrigation and fertilization on vertical distribution of soil NO_3_^−^-N content in root zone soil of different treatment at 43 days after transplanting in 2012. W1: 100%  ET_0_, W2: 75%  ET_0_, and W3: 50%  ET_0_; F1: N240–P_2_O_5_120–K_2_O150 kg hm^−2^; F2: N180–P_2_O_5_90–K_2_O112.5 kg hm^−2^; and F3: N120–P_2_O_5_60–K_2_O75 kg hm^−2^.

The vertical distribution of soil NO_3_^−^-N contents in root zone of different irrigation and fertilizer treatments at 43 days after transplanting is shown in Figure 4. In 43 days after transplanting, the soil NO_3_^−^-N contents ranged from 12.03 to 120.72 mg kg^−1^, which was higher than 25 days after transplanting, overall. There was a symmetrical distribution of soil NO_3_^−^-N contents in W1 treatment, and the axis of symmetry was located on the center of emitter. Increasing level of irrigation amount, the soil NO_3_^−^-N contents in soil profile showed obvious trend of moving far and down to deep soil layers. Surprisingly, the migration speed of soil NO_3_^−^-N in horizontal direction was higher than vertical direction, and the accumulation area appeared on the upper layer and around 20 cm (Figure 4(a)). The soil NO_3_^−^-N content was increased along the horizontal direction and decreased along the vertical direction in W2 treatment (Figure 4(b)). In F1 and F2 treatments, a large amount of soil NO_3_^−^-N was distributed in 15−30 cm, but the soil NO_3_^−^-N content of F3 treatment was reduced with the increase of soil depth (Figure 4(c)). In W3 treatment, there is a positive relationship between the soil NO_3_^−^-N content and the N fertilizer application rate, and the vertical distribution of soil NO_3_^−^-N contents in root zone was similar to W1 treatment.

**Figure 5:**
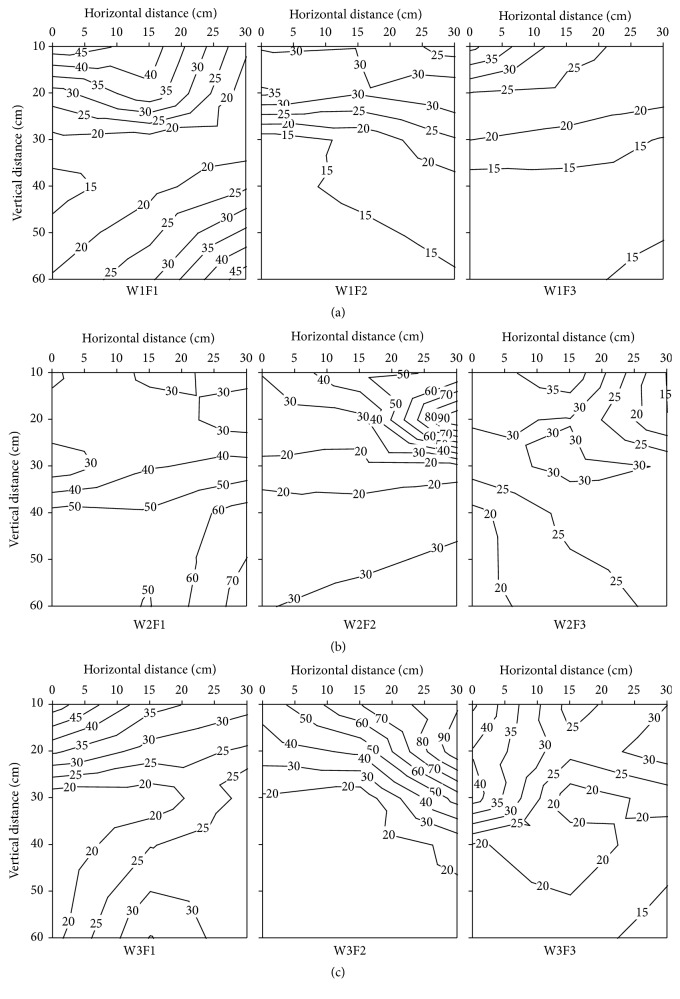
The effects of different irrigation and fertilization on vertical distribution of soil NO_3_^−^-N content in root zone soil of different treatment at 59 days after transplanting in 2012. W1: 100%  ET_0_, W2: 75%  ET_0_, and W3: 50%  ET_0_; F1: N240–P_2_O_5_120–K_2_O150 kg hm^−2^; F2: N180–P_2_O_5_90–K_2_O112.5 kg hm^−2^; and F3: N120–P_2_O_5_60–K_2_O75 kg hm^−2^.

The vertical distribution of soil NO_3_^−^-N contents in root zone of different irrigation and fertilizer treatments at 59 days after transplanting is shown in Figure 5. The average of soil NO_3_^−^-N content in 59 days after transplanting was lower than 43 days after transplanting. In W1 treatment, the soil NO_3_^−^-N accumulation area moves down to the center of the root system in F1, but the soil NO_3_^−^-N content in F2 was decreased with increase of soil depth. The result indicated that the higher the soil NO_3_^−^-N contents, the nearer the emitter in W1F3 treatment (Figure 5(a)). In W2 treatment, there was a positive correlation between the soil NO_3_^−^-N content and soil depth in F1 treatment; the opposite result is in F3 treatment. There was an accumulation area of soil NO_3_^−^-N content in 20 cm below the root zone in F2 treatment (Figure 5(b)). The further away the emitter, the lower the soil NO_3_^−^-N content in F1 and F3 treatment, but F2 has the opposite result and there is a high accumulation area at topsoil layers of 10−20 cm (Figure 5(c)).

**Figure 6:**
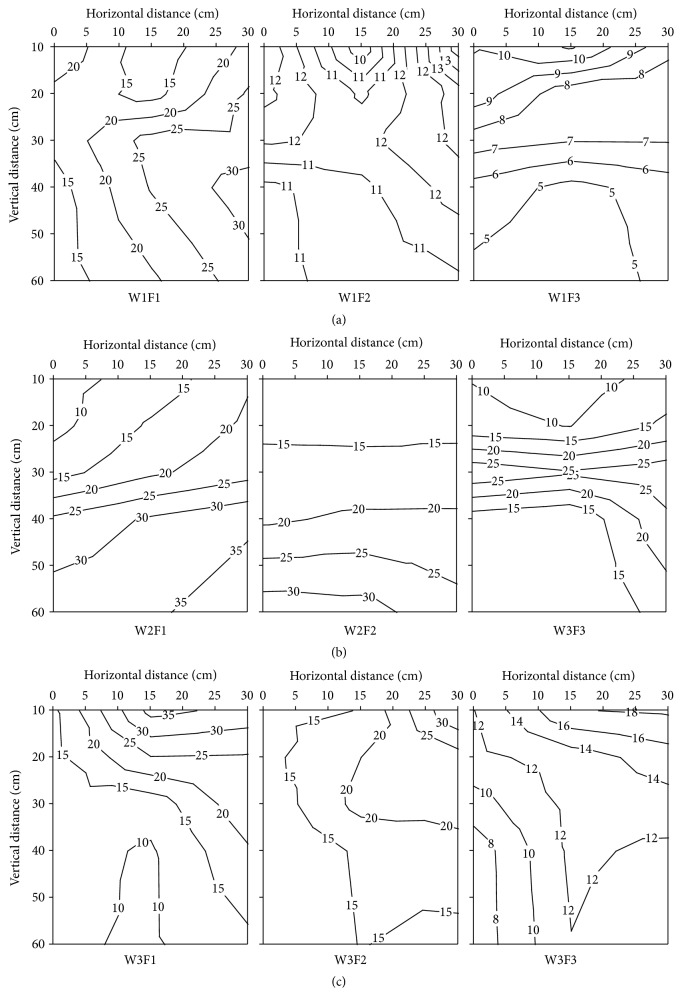
The effects of different irrigation and fertilization on vertical distribution of soil NO_3_^−^-N content in root zone soil of different treatment at 117 days after transplanting in 2012. W1: 100%  ET_0_, W2: 75%  ET_0_, and W3: 50%  ET_0_; F1: N240–P_2_O_5_120–K_2_O150 kg hm^−2^; F2: N180–P_2_O_5_90–K_2_O112.5 kg hm^−2^; and F3: N120–P_2_O_5_60–K_2_O75 kg hm^−2^.

The vertical distribution of soil NO_3_^−^-N contents in root zone of different irrigation and fertilizer treatments at 117 days after transplanting is shown in Figure 6. In the whole growth period, the lowest soil NO_3_^−^-N content appears at 117 days after transplanting, and the soil NO_3_^−^-N content ranged from 4.11 to 35.68 mg kg^−1^ and the values were increased with the increase of N fertilizer input. However, the values were increased first and then they go down with increasing the irrigation amount. In vertical direction, the soil NO_3_^−^-N content was increased from 20 cm to 60 cm in F1 and F2 treatment. There was a negative correlation between soil NO_3_^−^-N content and the horizontal distance from the emitter. The average soil NO_3_^−^-N content in 0−60 cm was significantly affected by the interaction of irrigation amount and soil depth. In contrast to the response of soil NO_3_^−^-N content to irrigation management, statistics do show a positive correlation between soil NO_3_^−^-N content and N fertilizer application levels.

Statistical analysis of soil NO_3_^−^-N content in 0−60 cm on irrigation and N fertilizer input levels is shown in Table 2; the result indicated that the soil NO_3_^−^-N content declined with the reduce of N fertilizer application rate. The highest of average soil NO_3_^−^-N content occurred in W2F1 treatment, which was 6.29% higher than the W1F1 treatment in 2012. Irrigation has significantly influenced the soil NO_3_^−^-N content in 0−60 cm in both years, and the fertilizer has greatly significantly influenced soil NO_3_^−^-N content. The great variation character of soil NO_3_^−^-N content has occurred at W3F1 treatment, and the coefficient of variation was 0.48 and 0.49 in 2012 and 2013, respectively. The coefficient ranged from 0.09 to 0.49 in two years. The interaction of irrigation and fertilizer was significantly affected by the soil NO_3_^−^-N content in 0−60 cm soil profile.

### 3.3. Fruit Yield, Nitrogen Recovery Efficiency, and Use Efficiency

**Figure 7:**
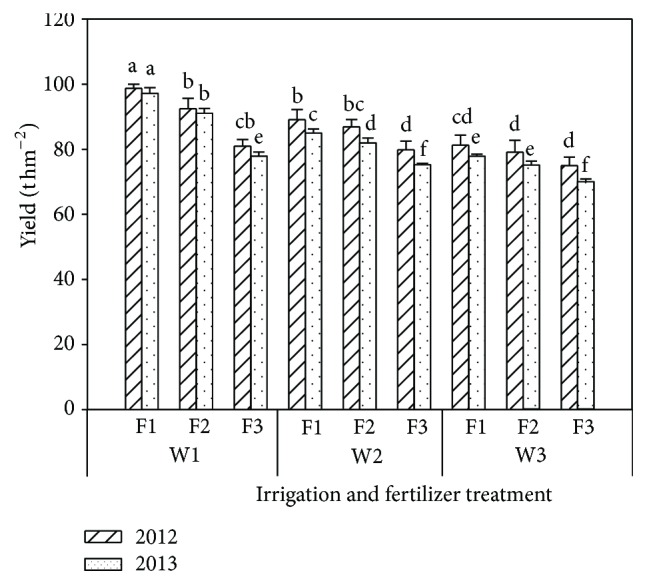
Effects of different irrigation and fertilizer treatments on tomato yield in the 2012 and 2013 seasons. Columns with the same letter represent values that are significant at the 5% probability level. Each value is the mean ± SD (*n* = 3).

The economic benefit of tomatoes is determined by the fruit yield and quality. Both water and fertilizer are essential factors for tomato growth and influence fruit yield. The relationships between fruit yield and different irrigation and fertilizer treatment are shown in Figure 7. The interactions between irrigation and N fertilizer were important for fruit yield and the single factors of irrigation or fertilizer were very significant for fruit yield. The fruit yield ranged from 70.6 to 97.8 t hm^−2^, and there was a positive correlation between fruit yield and irrigation and between fruit yield and fertilization. The difference in average fruit yield between F1 and F2 was small, but it was significantly greater than the F3 treatment. The fruit yield decreased with the irrigation amount reduce and ranged from 6.33 to 27.34% in 2012 and from 7.58 to 22.48% in 2013. The average fruit yields of the W2 and W3 treatments were 10.52% and 16.03% lower than W1, respectively. The tomato yield decreased with reduced fertilizer. The average tomato yields of the F2 and F3 treatments were 4.55% and 15.47% lower than F1 in 2012, respectively. The average tomato yields of the F2 and F3 treatments were 3.93% and 12.39% lower than F1 in 2013, respectively.

The N recovery efficiency and use efficiency after the harvest in two consecutive years are shown in Table 3. The N absorption content by plant was increased with the increase of N fertilizer rate and irrigation amount, but the N recovery efficiency was decreased with the increase of irrigation amount. The highest N absorption content of plant was 161.83 kg hm^−2^ in 2012, which was 74.25% higher than control treatment. However, the highest N use efficiency was obtained in W1F3 treatment, and the value of N use efficiency was 63.48% and 56.26% in 2012 and 2013, respectively. The lower the N fertilizer input, the higher the N use efficiency. In the same irrigation treatment, the average N use efficiency was increased with the increase of irrigation amount. Statistics do show a positive correlation between N recovery efficiency and N fertilizer application rates. The lower irrigation amount and the higher N recovery efficiency were obtained at the same N fertilizer application except F3 treatment.

## 4. Discussion

The large proportion of tomato root was distributed in the topsoil of 40 cm and rapidly decreased with the increase of soil depth [20], which can effectively absorb part of the soil nutrition in the root zone. Consequently, we have chosen the soil depth from 0 to 60 cm in this study. The results indicated that the soil NO_3_^−^-N content was increased with the increase of soil depth from 0 to 30 cm in 25 days after transplanting. The high-concentration soil NO_3_^−^-N content was mainly distributed at the topsoil layers (0−20 cm) in 43 days after transplanting, and the soil NO_3_^−^-N content was to reach 71.98 mg kg^−1^. The same result was obtained that the soil NO_3_^−^-N concentration was reduced from 30 cm. The most parts of soil NO_3_^−^-N content stayed in the topsoil (0–50 cm), which was clearly correlated to several root parameters [13, 21].

The results indicated that the weak correlation between N fertilizer application and N absorption contents and the vertical distribution of soil NO_3_^−^-N content in topsoil layer is characterized by rapid change under different N fertilizer levels. However, in this study, the soil NO_3_^−^-N content in W1F1 treatment was increased continuously from 30 to 60 cm at 59 days after transplanting; this phenomenon may be related to the N transferring quantity in the subsoil that is determined by the levels of irrigation and N fertilizer. With the increase of soil depth in W1 treatment, the soil NO_3_^−^-N concentration first increases and then decreases [22].

The vertical distribution of soil NO_3_^−^-N content ranged from 7.29 to 71.98 mg kg^−1^ on 25 days after transplanting. The high-concentration area of soil NO_3_^−^-N content was present in the upper soil layer (25–30 cm) in F1 treatment and the topsoil (15–20 cm) in F3 treatment. The same results were reported that the peak soil NO_3_^−^-N concentration in 15–30 cm was observed in deficit irrigation and in 30–60 cm in full irrigation treatments [23]. The accumulation area of soil NO_3_^−^-N contents was mainly distributed below the root system; that is, the highest concentration of soil NO_3_^−^-N content was found in 30 cm away from the emitter in horizontal direction. It is surprising that the accumulation area of soil NO_3_^−^-N contents was also located in 30 cm away from the emitter in vertical and horizontal direction in W1F1 treatment. The reason may be the soil NO_3_^−^-N and soil water were moving synchronously. A similar trend for soil NO_3_^−^-N contents distribution values in response to high levels of water and fertilizer input is reported by Fang et al. [24] for wheat-maize double cropping system under fertilization and irrigation regimes in the North China Plain [24].

However, the soil NO_3_^−^-N content in horizontal direction was higher than vertical direction at the same distance away from the emitter; the reason may be the higher soil water content below the emitter which may reduce aeration and the partial anaerobic environment occurs at the same time [25]. The levels of irrigation were significantly affected by soil NO_3_^−^-N content. The fertilizer nitrogen rate was significantly influenced by soil NO_3_^−^-N content. The coefficient variation was increased with the irrigation amount decrease. The results indicate that the effect of coefficient variation on soil NO_3_^−^-N content was related to the level of irrigation and fertilization.

Approximately 30–45% of the applied fertilizer N was residual in the soil profiles; the higher N recovery efficiency does not mean the higher N use efficiency. The reason may be the fact that part of residual N in soil would be reused in the next growing season. Hence, the most effective way to improve the fruit yield is to reduce the N fertilizer losses. Similar results were reported that where soil NO_3_^−^-N accumulation was greater in cropping systems with greater N fertilizer input, it was surprisingly insensitive to differences in harvested N output [26, 27]. The higher N recovery efficiency was related to the higher fruit yield. Moreover, it means improvements in N use efficiency such as a better nutrient balance to counterdepletion of soil nutrients and soil acidification and better application techniques to improve nutrient uptake and to reduce nutrient losses. The similar result was obtained that N use efficiency for cereal production is approximately 33%, and the unaccounted portion represents loss of fertilizer N which from gaseous plant emission, soil denitrification, surface runoff, volatilization, and leaching [28].

## 5. Conclusion

The result demonstrates that wetting front transport distance is going to be identical in the irrigation levels. The negative correlation between N use efficiency and levels of N fertilizer application shows that the N recovery efficiency was increased with the increase of N fertilizer. In view of the various factors residual N and N absorption contents by plant, W1F2 is recommended because it increased N use efficiency and higher nitrogen absorption contents by plant than other treatments except W1F1 treatment.

## Figures and Tables

**Table 1:** The contents of organic matter, available N, available P, and available K of experimental soil.

Soil layer (cm)	Particle size < 2 mm (%)	Bulk density (g cm^−3^)	Organic matter (g kg^−1^)	Total N (mg kg^−1^)	Nitrate N (mg kg^−1^)	Available P (mg kg^−1^)	Available K (mg kg^−1^)
0–30	19.7	1.13	16.2	0.97	5.8	0.66	130.4
30–60	52.5	1.22	14.1	0.88	9.7	0.52	141.7
60–90	42.1	1.28	11.7	0.54	4.9	0.41	117.8

**Table 2:** Statistical analysis of soil NO_3_^−^-N content from 0 to 60 cm soil depth was analyzed on different irrigation and fertilization treatment.

Treatment	2012			2013		
	Mean value	Standard deviation	CV	Mean value	Standard deviation	CV
W1F1	21.00	6.63	0.32	26.25	7.12	0.31
W1F2	11.91	1.05	0.09	13.24	1.18	0.09
W1F3	6.99	2.09	0.12	7.13	2.11	0.15
W2F1	22.41	9.88	0.44	32.57	10.36	0.48
W2F2	18.91	6.76	0.36	17.68	6.50	0.41
W2F3	15.59	7.38	0.37	15.33	7.21	0.37
W3F1	17.96	8.69	0.48	18.24	8.88	0.49
W3F2	17.28	6.33	0.37	16.58	6.39	0.39
W3F3	12.36	3.30	0.27	11.37	3.14	0.23
Irrigation	0.019^*∗*^	—	—	0.023^*∗*^	—	—
Fertigation	0.0001^*∗∗∗*^	—	—	0.001^*∗∗∗*^	—	—
Irrigation × fertigation	0.014^*∗*^	—	—	0.008^*∗∗*^	—	—

The values are statistically significant by Duncan's test at *P* < 0.05. ^*∗*,*∗∗*,*∗∗∗*^Significant at *P* ≤ 0.05, 0.01, and 0.001 level, respectively, where CV is coefficient of variation.

**Table 3:** The fertilizer N recovery efficiency and use efficiency were after harvest in two consecutive years.

Treatment	2012				2013			
	Effective residual N (kg hm^−2^)	N absorption contents (kg hm^−2^)	N recovery efficiency (%)	N use efficiency (%)	Effective residual N (kg hm^−2^)	N absorption contents (kg hm^−2^)	N recovery efficiency (%)	N use efficiency (%)
W1F1	82.38	161.83	27.35%	50.25	91.21	163.41	31.68%	50.07
W1F2	64.45	147.02	18.27%	58.77	71.65	144.50	20.87%	56.25
W1F3	66.09	117.41	4.10%	63.48	70.41	110.76	2.16%	56.26
W2F1	110.25	136.06	28.22%	39.51	120.88	141.15	34.77%	40.79
W2F2	93.03	127.30	23.19%	47.82	86.98	126.82	19.57%	46.43
W2F3	86.70	95.44	2.97%	45.18	85.25	95.36	1.70%	43.43
W3F1	150.13	121.08	38.60%	33.27	152.47	103.56	32.27%	25.13
W3F2	144.45	107.82	40.94%	36.99	138.60	97.34	31.87%	30.05
W3F3	103.32	85.62	8.63%	36.99	97.04	83.52	1.65%	33.56
Control		41.23				43.25		
Initial N content	137.35				143.58			
